# Oxidized Low-Density Lipoprotein Contributes to Atherogenesis via Co-activation of Macrophages and Mast Cells

**DOI:** 10.1371/journal.pone.0123088

**Published:** 2015-03-26

**Authors:** Chong Chen, Damir B. Khismatullin

**Affiliations:** Department of Biomedical Engineering, Tulane University, New Orleans, Louisiana, United States of America; University of Michigan, UNITED STATES

## Abstract

Oxidized low-density lipoprotein (OxLDL) is a risk factor for atherosclerosis, due to its role in endothelial dysfunction and foam cell formation. Tissue-resident cells such as macrophages and mast cells release inflammatory mediators upon activation that in turn cause endothelial activation and monocyte adhesion. Two of these mediators are tumor necrosis factor (TNF)-α, produced by macrophages, and histamine, produced by mast cells. Static and microfluidic flow experiments were conducted to determine the number of adherent monocytes on vascular endothelium activated by supernatants of oxLDL-treated macrophages and mast cells or directly by oxLDL. The expression of adhesion molecules on activated endothelial cells and the concentration of TNF-α and histamine in the supernatants were measured by flow cytometry and enzyme-linked immunosorbent assay, respectively. A low dose of oxLDL (8 μg/ml), below the threshold for the clinical presentation of coronary artery disease, was sufficient to activate both macrophages and mast cells and synergistically increase monocyte-endothelium adhesion via released TNF-α and histamine. The direct exposure of endothelial cells to a much higher dose of oxLDL (80 μg/ml) had less effect on monocyte adhesion than the indirect activation via oxLDL-treated macrophages and mast cells. The results of this work indicate that the co-activation of macrophages and mast cells by oxLDL is an important mechanism for the endothelial dysfunction and atherogenesis. The observed synergistic effect suggests that both macrophages and mast cells play a significant role in early stages of atherosclerosis. Allergic patients with a lipid-rich diet may be at high risk for cardiovascular events due to high concentration of low-density lipoprotein and histamine in arterial vessel walls.

## Introduction

75% of all cardiovascular-related deaths in the United States are linked to atherosclerosis [1], a progressive disorder of medium- to large-size arteries characterized by the formation and calcification of atheromatous plaques in the arterial vessel walls. Atherosclerosis is recognized as a chronic inflammatory condition that begins with the dysfunction or activation of arterial endothelium. The increased expression of adhesion molecules on the surface of activated endothelial cells leads to a large number of monocytes attached to the endothelium. These adherent cells eventually transmigrate through the endothelium and accumulate in the intimal layer of the artery wall, where they differentiate into macrophages and, in the presence of certain factors, into foam cells [2]. The foam cells are the main component of a fatty streak [3].

Several lines of evidence indicate that low-density lipoprotein (LDL) and especially its oxidized form (oxLDL) play a key role in endothelial dysfunction and atherogenesis [4, 5]. LDL can be oxidized by vascular endothelial cells, smooth muscle cells or macrophages [6]. OxLDL binds to its lectin-like receptor LOX-1 in endothelial cells [7, 8] and triggers the CD40/CD40L signaling pathway [9], which in turn leads to the synthesis of chemokines [10, 11] and cell adhesion molecules [12, 13] involved in the adhesion of monocytes to the endothelium. Monocytes and macrophages uptaking oxLDL via scavenger receptors [14] release tumor necrosis factor-α (TNF-α) [15]. The uptake of oxLDL by macrophages transform these cells into foam cells [2].

Mast cells, which play a crucial role in allergy by releasing histamine during their degranulation [16], have been found in increased numbers near atherosclerotic lesions [17]. In the earliest stage of atherosclerosis, they are preferentially located in the adventitial layer of the artery wall, but they migrate closer to intimal macrophages at later stages, where they help convert macrophages into foam cells [18] and, together with macrophages, degrade extracellular matrix proteins in the shoulder regions of the atherosclerotic plaque [19]. The latter activity makes the plaque unstable or vulnerable, eventually causing thromboembolic events often resulting in a heart attack or stroke. Histamine released from degranulated mast cells is the primary mediator of allergic inflammation, but it also can increase the vascular wall permeability for LDL and promote the atherosclerotic lesion formation [20]. Histamine causes the proliferation of smooth muscle cells and their migration to the lesion [21]. The systemic activation of mast cells increases the plaque progression and, in advanced atherosclerosis, leads to the intraplaque hemorrhage due to the release of histamine [22].

The LDL particles are 21–27 nm in diameter [23], i.e., they are small enough to transport through the artery wall. According to in vivo measurements [24], they are accumulated in both the intimal and adventitial layers of the wall. When oxidized, they come in contact with and activate intimal macrophages, which then release TNF-α [15]. Later on, some of the oxLDL particles move to the adventitia, where they interact with adventitial mast cells. The current knowledge is that mast cells can be activated by oxLDL-IgG immune complexes [25]. However, oxLDL can sensitize mast cells even without complexing with IgG molecules [26]. We hypothesize that LDL, oxidized in the artery wall, co-activates macrophages and mast cells, which in its turn leads to the release of TNF-α and histamine that have a sequential and synergistic effect on endothelial dysfunction and monocyte adhesion. It is important to say that, in the scenario described above, vascular endothelial cells are first exposed to TNF-α from activated intimal macrophages and then to histamine from activated adventitial mast cells. We test the hypothesis in static and microfluidic flow adhesion experiments and by enzyme-linked immunosorbent assays (ELISA) and flow cytometry.

## Materials and Methods

This study was performed without using human or animal subjects.

### LDL oxidation

Human LDL was purchased from Kalen Biomedical (Montgomery Village, MD). The LDL was oxidized according to the protocol of Huang et al. [27]. Briefly, LDL (2 mg/ml) was incubated with 10 μmol/l copper sulfite (CuSO_4_) at 37°C for 18 hours. The copper ions were removed by using Amicon Ultra Centrifugal Filters (Millipore, Billerica, MA) with a molecular weight cutoff of 100 KDa. EDTA (300 μmol/l) was added immediately after the filtration procedure to prevent any further oxidation. The degree of LDL oxidation was determined by the thiobarbituric acid-reactive substances (TBARS) assay. The TBARS assay measures malondialdehyde (MDA), which is the end product of lipid peroxidation. The reaction of thiobarbituric acid with MDA forms a product that emits light at wavelength of 585 nm. The MDA content in the oxLDL samples was determined by measuring the fluorescence intensity with a microplate reader SpectraMAX Gimini EM (GMI, Ramsey, MN). The TBARS assay showed that the MDA concentration increased threefold with the copper ion treatment of LDL: from 7.40±0.13 nmol/mg in LDL to 30.85±1.12 nmol/mg in copper ion-treated LDL. This confirmed that LDL transformed into oxLDL [27].

### Cell culture

Primary human umbilical vein endothelial cells (HUVEC, pooled) were purchased from Invitrogen/Cascade Biologics. The HUVEC growth medium was Medium 200 (Invitrogen) with Low Serum Growth Supplement (LSGS, Invitrogen) and Gentamicin/Amphotericin B (Invitrogen). HUVEC of passage 3–5 were used in all experiments. The cell adhesion experiments were conducted after endothelial cells reached confluence. It should be noted that 1) venous and arterial endothelial cells have a similar expression pattern of adhesion molecules when they are activated by TNF-α and oxLDL [12, 13, 28, 29], and 2) HUVEC are a well-established model for the analysis of endothelial dysfunction in atherosclerosis [13, 27, 30, 31].

THP-1, a human acute monocytic leukemia cell line, was purchased from ATCC (Manassas, VA). THP-1 cells were grown in RPMI-1640 medium (ATCC), supplemented with 10% fetal bovine serum (Invitrogen), 1% Penicillin/Streptomycin (Invitrogen) and 0.05 mmol/l 2-mercaptoethanol (Sigma Aldrich). THP-1 cells were differentiated into macrophages by incubating with 10 ng/ml phorbol 12-myristate 13-acetate (PMA, Sigma-Aldrich) for 48 hours. 10 ng/ml PMA led to 99.3% ± 0.3% of differentiated cells, in agreement with Park et al. [32]. THP-1 cells resemble primary monocytes/macrophages and mimic the alteration of macrophages in atherosclerotic lesions [33]. Our recent investigation [34] showed the closest similarity between THP-1 cells and monocytes isolated from human peripheral blood mononuclear cells (PBMC) in their adhesion to vascular endothelium.

Human mast cells (LUVA cell line) were the generous gift of Dr. John Steinke (Division of Allergy and Immunology, University of Virginia). They were obtained from CD34-positive enriched mononuclear cells derived from a donor with aspirin-exacerbated respiratory disease [35]. The LUVA cells were maintained at a concentration of 5×10^5^ cells/ml according to the protocol of Laidlaw et al. [35]. The complete growth medium for LUVA cells was StemPro-34 SFM (Invitrogen) with StemPro-34 nutrient supplement (Invitrogen), 1% penicillin/streptomycin (Invitrogen) and 1% L-glutamine-200mM (Invitrogen).

All cells in this study were maintained in tissue culture flasks or BD Falcon 96-well plates (Becton Dickinson, Franklin Lakes, NJ) in a 37°C, 5% CO_2_ incubator.

### ELISA measurements and HUVEC activation groups

THP-1 monocytes and THP-1 macrophages were incubated with 8 μg/ml oxLDL for 20 hours. This concentration corresponds to the lower limit of serum measurements in patients with atherosclerosis [36, 37]. The concentration of TNF-α in the supernatant of oxLDL-activated THP-1 cells or macrophages was measured by a TNF-α ELISA kit (Invitrogen). Specifically, the supernatant of THP-1 cells or macrophages, exposed or not to OxLDL, was added to the wells coated with the anti-TNF-α antibody. After a two-hour incubation at room temperature, the horseradish peroxidase (HRP)-labeled anti-TNF-α antibody was added to the wells to form a sandwich: well surface—antibody—TNF-α—antibody—HRP. After antibody-antigen reactions occurred, the excess of antigens (or antibodies) was removed by washing the wells three times. The labeled antibodies were measured by adding chromogen (tetramethylbenzidine, TMB) and reading absorbance at 450 nm using the SpectraMAX Gimini XS microplate reader (GMI, Ramsey, MN).

LUVA mast cells were centrifuged and resuspended in RPMI-1640 basal medium, RPMI-1640 basal medium containing 8 or 25 μg/ml of oxLDL, or the supernatant of oxLDL-treated macrophages for three hours. After incubation, the supernatants of mast cells were collected, and the histamine level in each supernatant was measured by a histamine ELISA kit (Eagle Biosciences, Nashua, NH) using the SpectraMAX Gimini XS microplate reader.

**Fig 1:**
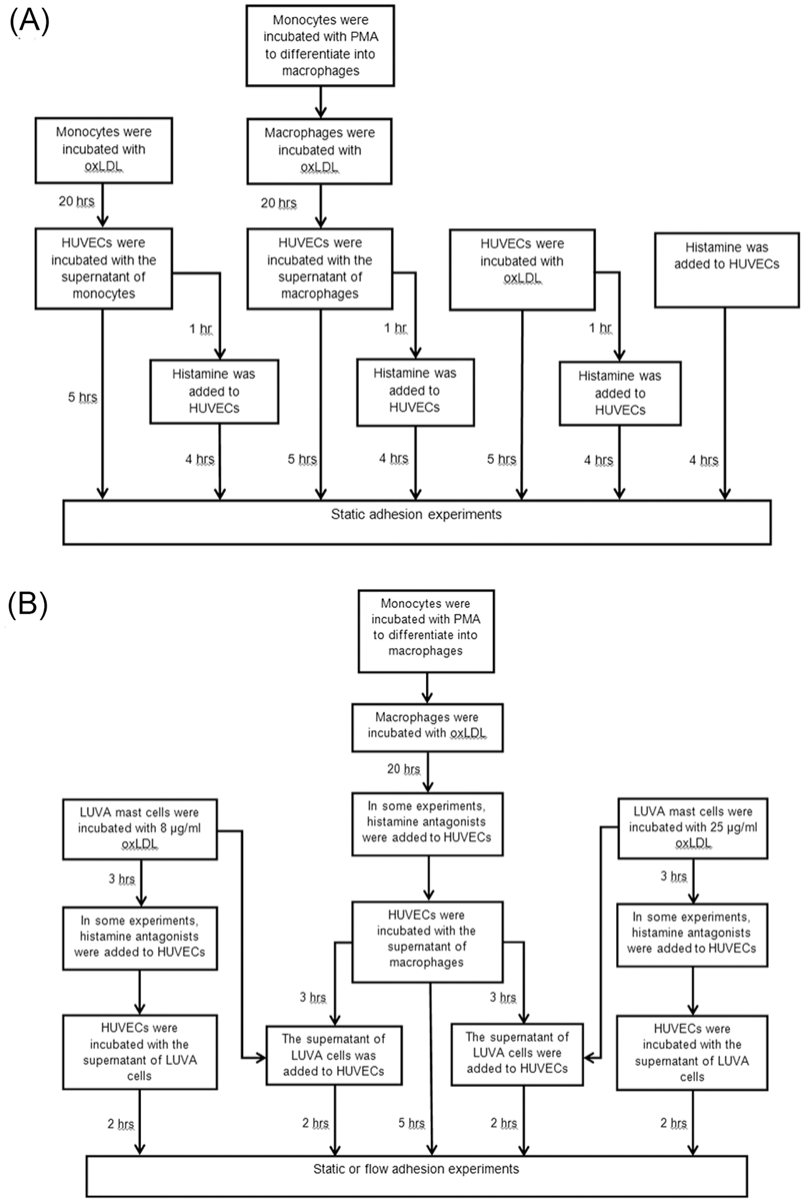
(A) Flow chart of static adhesion experiments in which HUVECs were exposed to oxLDL, histamine, and/or the supernatant of oxLDL-treated monocytes or macrophages. (B) Flow chart of static and flow adhesion experiments in which HUVECs were exposed to the supernatant of oxLDL-treated macrophages and/or the supernatant of oxLDL-treated mast cells.

In the first set of experiments, the supernatant of 8 μg/ml oxLDL-treated THP-1 cells (S_T_) and the supernatant of 8 μg/ml oxLDL-treated macrophages (S_M_) were collected. To investigate the combined effect of the supernatant and histamine on the adherence of THP-1 monocytes to vascular endothelium, the following HUVEC activation groups were considered: 1) *control*—HUVEC incubated in RPMI-1640 basal medium; 2) *histamine*—HUVEC incubated in RPMI-1640 basal medium with 10^-6^ mol/l histamine for four hours; 3) *S*_*T*_—HUVEC incubated in S_T_ for five hours; 4) *S*_*T*_ *+ Hist*—HUVEC incubated in S_T_ for five hours with 10^-6^ mol/l histamine added four hours before the end of the incubation procedure; 5) *S*_*M*_—HUVEC incubated in S_M_ for five hours; and 6) *S*_*M*_ *+ Hist*—HUVEC incubated in S_M_ for five hours with 10^-6^ mol/l histamine added four hours before the end of the incubation procedure. Here, we also considered the direct activation of HUVEC by oxLDL and histamine: 7) *OxLDL*—HUVEC incubated in RPMI-1640 basal medium with 80 μg/ml oxLDL for 20 hours; and 8) *OxLDL + His*t—HUVEC incubated in RPMI-1640 basal medium with 80 μg/ml oxLDL for 20 hours and with 10^-6^ mol/l histamine added 4 hours before the end of the oxLDL incubation procedure. The flow chart of HUVEC activation for the first set of experiments is shown in Fig. 1A.

In the second set of experiments, LUVA cells were centrifuged and resuspended in RPMI-1640 basal medium with 8 μg/ml or 25 μg/ml oxLDL for three hours. After the incubation, the supernatants of 8 μg/ml and 25 μg/ml oxLDL-treated LUVA cells, denoted respectively as S_L1_ and S_L2_, were applied to HUVEC. Diphenhydramine (10^-5^ mol/L) and ranitidine (10^-5^ mol/L), which are the antagonists of histamine receptors H1 and H2, were used to determine whether the effect of the mast cell supernatants on monocyte-endothelium adhesion is caused by histamine. The HUVEC activation groups in these experiments were: 1) *control*—HUVEC in RPMI-1640 basal medium alone; 2) *S*_*L1*_—HUVEC incubated in S_L1_ for two hours; 3) *S*_*L1*_ *+ H* ^*-*^—HUVEC incubated in S_L1_ with the antagonists of histamine receptors for two hours; 4) *S*_*M*_—HUVEC incubated in S_M_ for five hours; 5) *S*_*M*_ *+ H* ^*-*^—HUVEC incubated in the S_M_ with the antagonists of histamine receptors for five hours; 6) *S*_*M*_ *+ S*_*L1*_—HUVEC incubated in S_M_ for three hours and then in S_L1_ for two hours; 7) *S*_*M*_ *+ S*_*L1*_ *+ H* ^*-*^—HUVEC incubated in the S_M_ with the antagonists of histamine receptors for three hours and then in S_L1_ for two hours; 8) *S*_*M*_ *+ S*_*L2*_—HUVEC incubated in S_M_ for three hours and then in S_L2_ for two hours; and 9) *S*_*M*_ *+ S*_*L2*_ *+ H* ^*-*^—HUVEC incubated in the S_M_ with the antagonists of histamine receptors for three hours and then in S_L2_ for two hours. Fig. 1B displays the flow chart of HUVEC activation for the second set of experiments.

To evaluate the TNF-α dose effect on the number of adherent THP-1 cells on HUVEC, TNF-α with a concentration from 0 to 10 ng/ml was applied to HUVEC for five hours. THP-1 cells (1×10^6^ cell/ml), stained with DiO fluorescence (Vybrant Cell-Labeling Solutions, Invitrogen) characterized by the emission wavelength of 501 nm, were added to HUVEC and incubated for 15 minutes. After the incubation, the THP-1 cell suspension was removed, and each well was washed three times with PBS to eliminate non-adherent cells. The HUVEC monolayer was visualized using a 10X objective in an inverted epifluorescence microscope (Nikon Eclipse TiS). Fluorescent images of firmly adherent THP-1 cells were taken by a digital CCD camera (Qimaging Retiga EXi) at three different areas of each well. The recorded images were processed by custom data analysis software to determine the number of adherent cells. The image field size was 904 μm × 675 μm.

### Shear flow-induced detachment assays

Flow adhesion assays were conducted using BioFlux 200 microfluidic shear flow system (Fluxion Biosciences, San Francisco, CA). HUVEC were seeded in the microchannels of a BioFlux 48-well plate, according to the protocol developed in our previous studies [34, 38]. One BioFlux 48-well plate contains 24 microchannels, each connecting one inlet and one outlet wells. The microchannels have a rectangular cross section with a height of 70 μm and a width of 350 μm. When HUVEC reached confluence in the viewing portions of the microchannels, they were activated by the supernatants of oxLDL-treated mast cells and/or oxLDL-treated macrophages, as described above. Following the HUVEC activation, the suspensions of THP-1 cells (1×10^6^ cell/ml) were perfused through the endothelium-coated channels with the wall shear stress (WSS) of 1.0 dyn/cm^2^. As the THP-1 cells entered the viewing portion of the channels, the WSS was reduced to 0.2 dyn/cm^2^ and maintained at this value for 15 minutes. Then, the WSS was increased to 0.6 dyn/cm^2^ (corresponding to the wall shear rate of 76 s^-1^), and the images of moving THP-1 cells in the channels were sequentially taken for five minutes with a frame rate of 5 frames per second.

### Fluorescence activated cell sorting (FACS) analysis

HUVEC grew to confluence in T-25 flasks, following the exposure to oxLDL-treated macrophage supernatant (S_M_) and then to histamine or the oxLDL-treated mast cell supernatant, as described above. Endothelial cells in each flask were first washed by ice cold PBS. Their detachment from the flask wall was achieved by exposing the cells to the Enzyme-Free PBS-based Cell Dissociation Buffer (Invitrogen) for 5 minutes. Fluorescein isothiocyanate (FITC)-conjugated mouse IgG1, mouse anti-human CD54 (Intercellular Adhesion Molecule 1, ICAM-1), CD62E (E-selectin) and CD106 (Vascular Cell Adhesion Molecules 1, VCAM-1) were added to HUVEC. All the antibodies were purchased from Ancell (Bayport, MN). The cells were incubated with antibodies on ice for 45 minutes, followed by washing with FACS buffer and resuspending in a buffer containing 2% formaldehyde. Flow cytometric analysis was conducted using the BD FACSCanto II system (Becton Dickinson).

### Statistical analysis

Three to four independent experiments per group were conducted and mean ± SD was shown in figures. The statistical significance was determined by one- or two-way ANOVA and Tukey's test.

## Results

### TNF-α release from oxLDL-treated monocytes and macrophages

**Fig 2:**
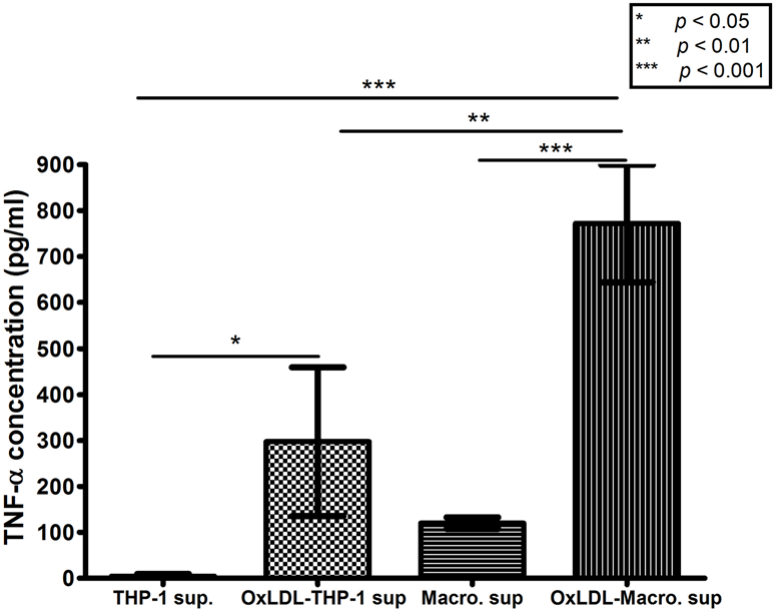
Concentration of TNF-α in the supernatants of untreated monocytes (“THP-1 sup”), untreated macrophages (“Macro. Sup”), 8 μg/ml oxLDL-treated monocytes (“OxLDL-THP-1 sup”), and 8 μg/ml oxLDL-treated macrophages (“OxLDL-Macro. Sup”), according to ELISA. Mean ± SD of three independent tests.

Fig. 2 shows the amount of TNF-α in the supernatants of THP-1 monocytes and macrophages exposed to 8 μg/ml oxLDL or left untreated, as measured by ELISA. OxLDL significantly increased the concentration of TNF-α released from monocytes (from 4.6 to 297.4 pg/ml; *p* < 0.05) and from macrophages (from 120.0 to 771.8 pg/ml; *p* < 0.001). Macrophages always release more TNF-α than monocytes.

### THP-1 cell adhesion to HUVEC activated by oxLDL, oxLDL-treated macrophages, and histamine

**Fig 3:**
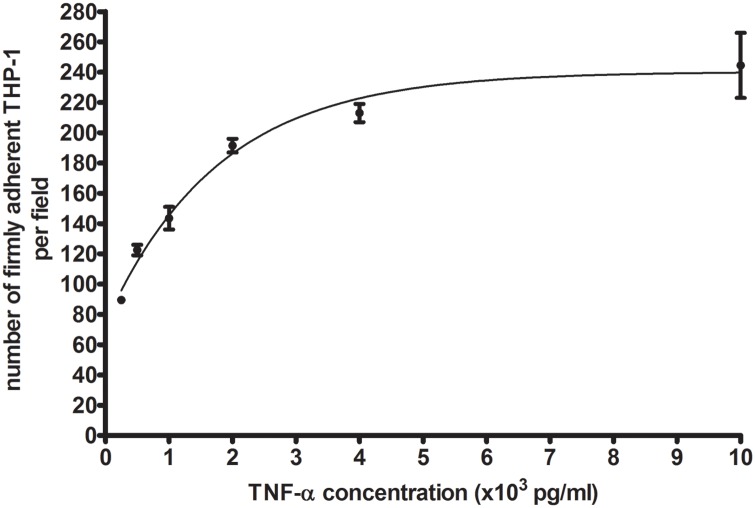
Number of adherent THP-1 cells on HUVEC activated by TNF-α with a concentration of 250, 500, 1000, 2000, 4000, or 10000 pg/ml. HUVEC were exposed to TNF-α for five hours. Mean ± SD per image field (904 μm × 675 μm) of three independent tests.

Besides TNF-α, oxLDL can induce THP-1 monocytes or macrophages to secrete a number of pro- and anti-inflammatory cytokines, such as IL-6, IL-8, and IL-10 [39–41]. To check whether TNF-α plays a dominant role in the monocyte adhesion to the monocyte/macrophage supernatant-activated endothelium, we first exposed HUVEC to different concentrations of TNF-α and measured the number of adherent THP-1 cells as a function of TNF-α concentration. The resulting titration curve, shown in Fig. 3, indicates that the TNF-α concentration of 297.4 pg/ml and 771.8 pg/ml, measured in the supernatants of oxLDL-treated THP-1 monocytes and macrophages (S_T_ and S_M_), corresponds to 103 and 141 adherent THP-1 cells, respectively.

**Fig 4:**
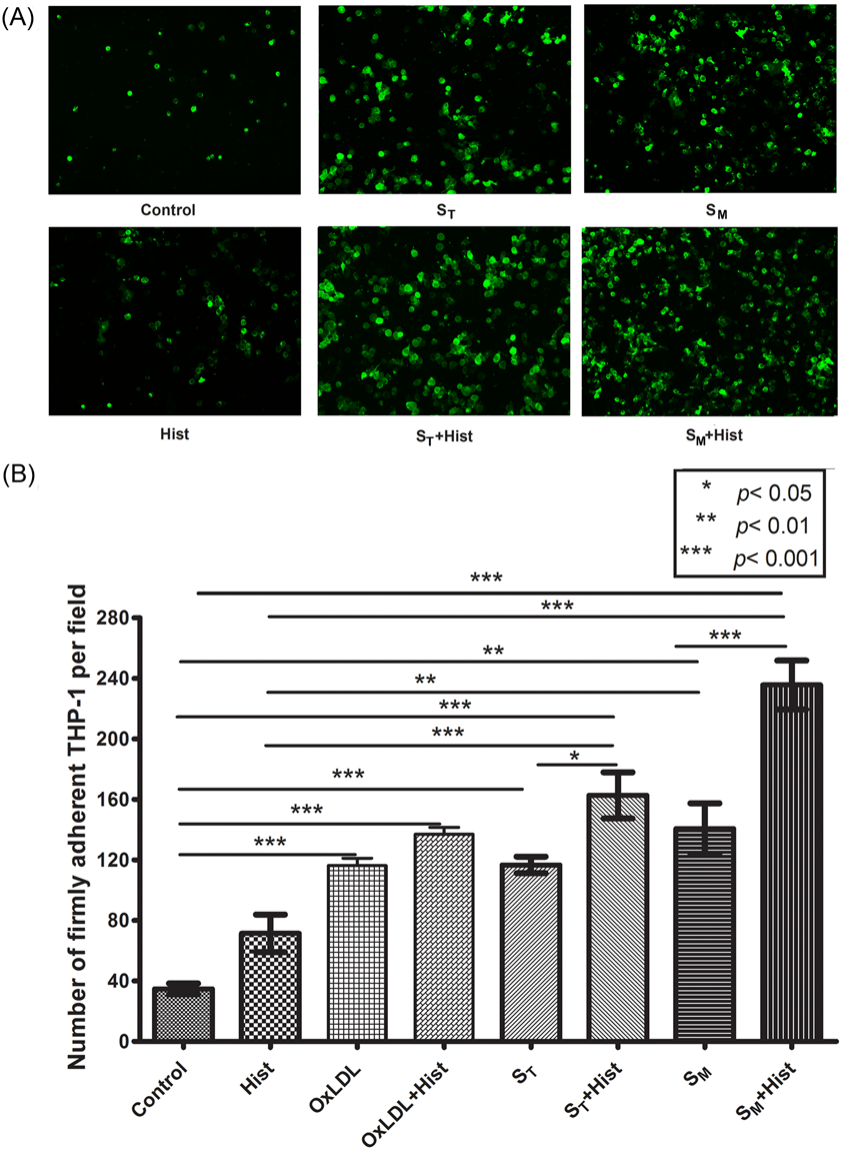
THP-1 cell adhesion to HUVEC exposed to oxLDL, histamine, or the macrophage supernatant. (A) Fluorescent images of adherent THP-1 cells (green) on resting (control) or activated HUVEC. (B) Number of adherent THP-1 cells (mean ± SD, n = 4) on resting or activated HUVEC per image field (904 μm × 675 μm). The HUVEC activation groups are: 1) “Control”—RPMI-1640 medium alone; 2) “Hist”—RPMI-1640 medium with 10^-6^ mol/l histamine for four hours; 3) “OxLDL”- 80 μg/ml of oxLDL for 20 hours; 4) “OxLDL + Hist”- 80 μg/ml of oxLDL for 20 hours with 10^-6^ mol/l histamine added 4 hours before the end of the incubation time; 5) “S_T_“—the supernatant of 8 μg/ml oxLDL-treated THP-1 cells for five hours; 6) “S_T_ + Hist”—S_T_ for five hours with 10^-6^ mol/l histamine added four hours before the end of the incubation time; 7) “S_M_“—the supernatant of 8 μg/ml oxLDL-treated THP-1 macrophages for five hours; and 8) “S_M_ + Hist”—S_M_ for five hours with 10^-6^ mol/l histamine added four hours before the end of the incubation time.

We then measured the number of adherent THP-1 cells on activated HUVEC under static conditions. As seen in Fig. 4, there were on average 117 and 141 adherent THP-1 cells in the case of S_T_ and S_M_ activation groups, respectively. When comparinng with the TNF-α titration curve, these data support that the THP-1 cell adhesion to the endothelium activated by oxLDL-treated macrophage supernatant is primarily mediated by TNF-α. Clearly, in the absence of TNF-α or at low TNF-α concentration, the endothelial dysfunction and THP-1 cell adhesion may be mediated by other pro-inflammatory cytokines released by activated monocytes or macrophages. This explains why there were more adherent THP-1 cells on endothelium exposed to the oxLDL-treated THP-1 monocyte supernatant than predicted by the TNF-α titration curve (117 vs 103, cf. Figs. 3 and 4). As discussed above, the TNF-α concentration was much less in the oxLDL-treated THP-1 monocyte supernatant than in the the oxLDL-treated THP-1 macrophage supernatant.

THP-1 cells, highlighted with green fluorescense, sequentially increased their adhesion to HUVEC in the control, Hist, S_T_, S_M_, S_T_+Hist, and S_M_+Hist groups (Fig. 4A). The control group had 35±8 adherent THP-1 cells (Fig. 4B). The histamine alone treatment increased this number to 72±20, while the activation of HUVEC with S_T_ and S_M_ significantly (*p*<0.01) increased the adherent cell population to 117±9 and 141±29, respectively. The additional exposure of S_T_- and S_M_-activated HUVEC to histamine led to a significantly (*p*<0.05) larger number of adherent THP-1 cells (163±26 and 236±27) than any single activation (S_T_, S_M_, or Hist) or control group.

When HUVEC was directly activated by oxLDL with a concentration of 80 μg/ml, the number of adherent THP-1 cells was 116±9 (Fig. 4B). This value is insignificantly different from (and even less than) the number of adherent cells in the S_T_ and S_M_ groups, where the oxLDL concentration was 10 times less (8 μg/ml). Moreover, histamine had much less effect on the endothelium directly exposed to 80 μg/ml oxLDL (137±7 adherent cells in the OxLDL + Hist group) than that on the endothelium exposed to the supernatant of 8 μg/ml OxLDL-treated macrophages (236±27 adherent cells in the S_M_+Hist group). These data show that oxLDL exerts its strong effect on vascular endothelium by activating tissue macrophages than by directly binding its receptors on endothelial cells.

**Fig 5:**
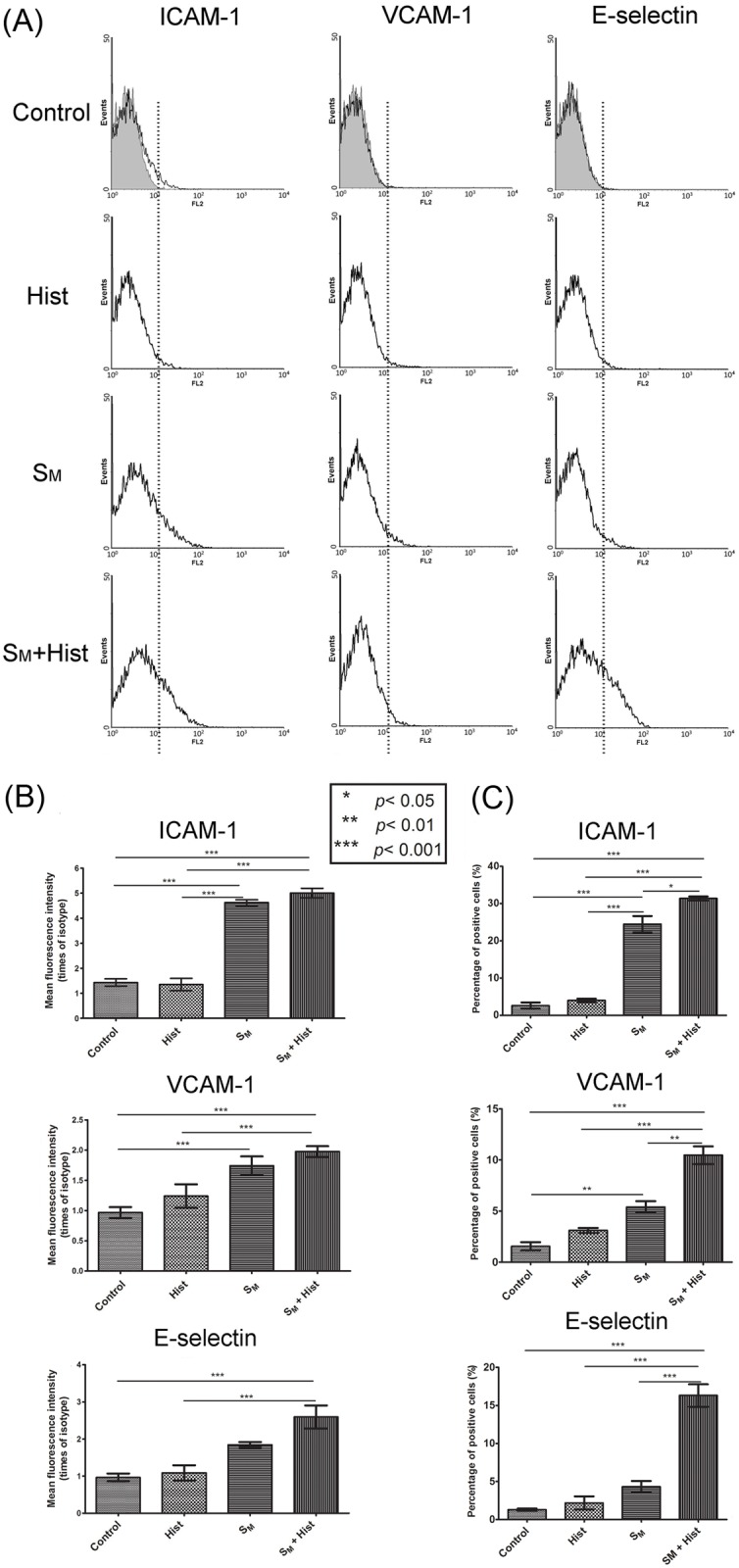
Surface expression of adhesion molecules on resting HUVEC (“Control”) and on HUVEC exposed to 10^-6^ mol/l histamine (“Hist”), the supernatant of 8 μg/ml oxLDL-treated macrophages (“S_M_”), or a combination of the oxLDL-treated macrophage supernatant and histamine (“S_M_+Hist”). (A) Histogram of the expression and (B) fluorescence intensity relative to the isotype control (mean ± SD, n = 3) of ICAM-1, VCAM-1 and E-selectin. (C) Percentage of HUVEC with positive expression of these adhesion molecules (mean ± SD, n = 3). Details about the HUVEC activation groups are given in Figs. 1 and 4.

The S_M_+Hist-activated HUVEC have the increased density of ICAM-1, VCAM-1 and E-selectin molecules on their surface, according to flow cytometry. In the histograms of these molecules (Fig. 5A), the peak shifted to the right in the S_M_+Hist activation group (green line), as compared to the control group (red line). According to the statistical analysis (Fig. 5B), the S_M_+Hist activation significantly (*p*<0.001) increased the fluorescence intensity (FI) of ICAM-1 (5.0±0.3), VCAM-1 (2.0±0.2) and E-selectin (2.6±0.5), as compared to the control group (ICAM-1: 1.4±0.2; VCAM-1: 1.0±0.2 and E-selectin: 1.0±0.2). The S_M_ alone activation strongly (*p* < 0.001) intensified the FI of ICAM-1 (4.6±0.2) and VCAM-1 (1.7±0.3), while histamine alone activation had no significant effect on the FIs of all three molecules.

In terms of the percentage of cells with positive expression of a specific adhesion molecule (Fig. 5C), the S_M_ activation group had 24.5% ± 3.7% of cells expressed ICAM-1, significantly (*p*<0.001) higher than the control group (2.6% ± 1.4%). Similarly, in the S_M_ group, there was a significant difference (*p*<0.01) in the percentage of cells with VCAM-1 (5.4% ± 1.2%), as compared to control (1.6% ± 0.7%). With the combined activation (S_M_+Hist), the percentage of cells positively expressed all three adhesion molecules (ICAM-1, VCAM-1, E-selectin) became significantly (*p* < 0.01) higher than that in the control group or in the groups with individual activation. Specifically, in the S_M_+Hist activation group, there were 31.4% ± 0.9% cells expressed ICAM-1, 10.5% ± 1.5% cells with VCAM-1, and 16.3% ± 2.6% cells with E-selectin (only 1.3% ± 0.3% control cells expressed E-selectin). TNF-α is known to induce the endothelial expression of ICAM-1, VCAM-1 and E-selectin [42], and it is likely responsible for the increased expression of these molecules on HUVEC activated by the oxLDL-treated macrophage supernatant (S_M_). The data in Fig. 5 also are in line with the results of our previous study, where TNF-α and histamine were shown to have a synergistic effect on the expression of endothelial ICAM-1, VCAM-1 and E-selectin [38].

### Histamine release from oxLDL-treated mast cells

**Fig 6:**
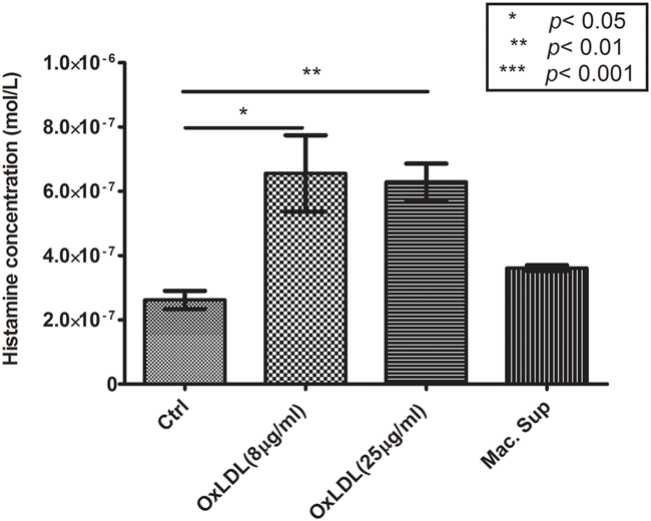
Concentration of histamine in the supernatants of untreated mast cells (“Ctrl”), 8 μg/ml oxLDL-treated mast cells (“OxLDL(8 μg/ml)”), 25 μg/ml oxLDL-treated mast cells (“OxLDL(25 μg/ml)”), and mast cells exposed to the supernatant of 8 μg/ml oxLDL-treated monocytes, according to ELISA. Mean ± SD of three independent tests.

Fig. 6 shows the histamine level in the supernatants of untreated mast cells (control), mast cells treated with 8 or 25 μg/ml oxLDL, and mast cells incubated with 8 oxLDL-treated macrophage supernatant (Mac. Sup). The concentration of histamine released by mast cells significantly (*p* < 0.05) increased from (0.26±0.03)×10^-6^ mol/l in the control group to (0.66±0.17)×10^-6^ mol/l or (0.63±0.06)×10^-6^ mol/l in the 8 μg/ml or 25 μg/ml OxLDL activation group, respectively. This is ~34% or ~37% less than the histamine concentration used in the first set of experiments (Figs. 4 and 5). The macrophage supernatant caused an insignificant increase in released histamine concentration to (0.36±0.01)×10^-6^ mol/l.

### THP-1 cell adhesion to HUVEC activated by oxLDL-treated macrophages and mast cells

**Fig 7:**
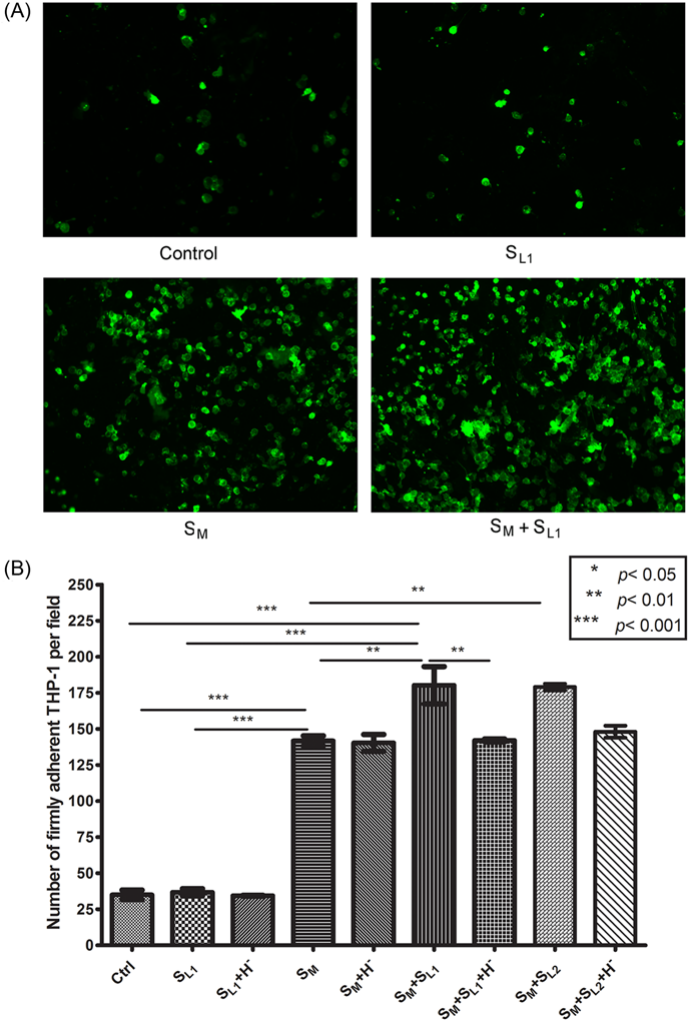
THP-1 cell adhesion to HUVEC exposed to the supernatants of oxLDL-treated macrophages and mast cells. (A) Fluorescent images of adherent THP-1 cells (green) on resting (control) and activated HUVEC. (B) Number of adherent THP-1 cells (mean ± SD, n = 4) on HUVEC per image field (904 μm × 675 μm). The HUVEC activation groups are: 1) “Control”—RPMI-1640 medium alone; 2) “S_L1_“—the supernatant of 8 μg/ml oxLDL-treated mast cells for two hours; 3) “S_L1_ + H^-^“—S_L1_ combined with the antagonists of histamine receptors for two hours; 4) “S_M_“—the supernatant of 8 μg/ml oxLDL-treated macrophages for five hours; 5) “S_M_ + H^-^“—S_M_ combined with the antagonists of histamine receptors for five hours; 6) “S_M_ + S_L1_“—S_M_ for five hours with S_L1_ added two hours before the end of the incubation time; 7) “S_M_ + S_L1_ + H^-^“—S_M_ combined with the antagonists of histamine receptors for five hours and with S_L1_ added two hours before the end of the incubation time; 8) “S_M_ + S_L2_“—S_M_ for five hours with the supernatant of 25 μg/ml oxLDL-treated mast cells (S_L2_) added two hours before the end of the incubation time; and 9) S_M_ + S_L2_ + H^-^—S_M_ combined with the antagonists of histamine receptors for five hours and with S_L2_ added two hours before the end of the incubation time.

Fig. 7A) shows the number of adherent THP-1 cells on HUVEC activated by oxLDL-treated macrophage (S_M_) and 8 μg/ml and 25 μg/ml oxLDL-treated mast cell supernatants (S_L1_ and S_L2_). The S_L1_ activation alone did not increase the THP-1 adhesion, as compared to control (37±3 adherent cells in the S _L1_ group vs. 35±5 in the control group). The exposure of HUVEC to S_M_ and S_M_ + S_L1_ significantly (*p*<0.001) elevated the number of adherent THP-1 cells from its control value to 142±7 and 180±22, respectively. The difference between individual exposures (S_M_ or S_L1_) and combined exposure (S_M_+S_L1_) also was statistically significant (*p*<0.01). Since S_L1_ alone had a negligible effect on HUVEC activation, these measurements indicate that the chemical mediators released from oxLDL-treated macrophages and mast cells synergistically increase the monocyte-endothelium adhesion. There was a negligible change in the number of adherent cells between the S_M_+S_L1_ and S_M_+S_L2_ activation groups (180±22 vs. 179±3), i.e., the mast cell activation level already reached a saturation point at 8 μg/ml of oxLDL. The observed synergistic effect of oxLDL-treated macrophages and mast cells was blocked by antagonists of histamine receptors H1 and H2 (*cf*. groups with H^-^ in Fig. 7B). The number of adherent cells was 142±3 in the S_M_+S_L1_+H^-^ group and 148±7 in the S_M_+S_L2_+H^-^ group, which were very close to the value obtained for the S_M_ alone activation (142±7). This result points out that histamine released from mast cells after oxLDL treatment is responsible for the increased adhesion of THP-1 cells to S_M_-activated HUVEC.

**Fig 8:**
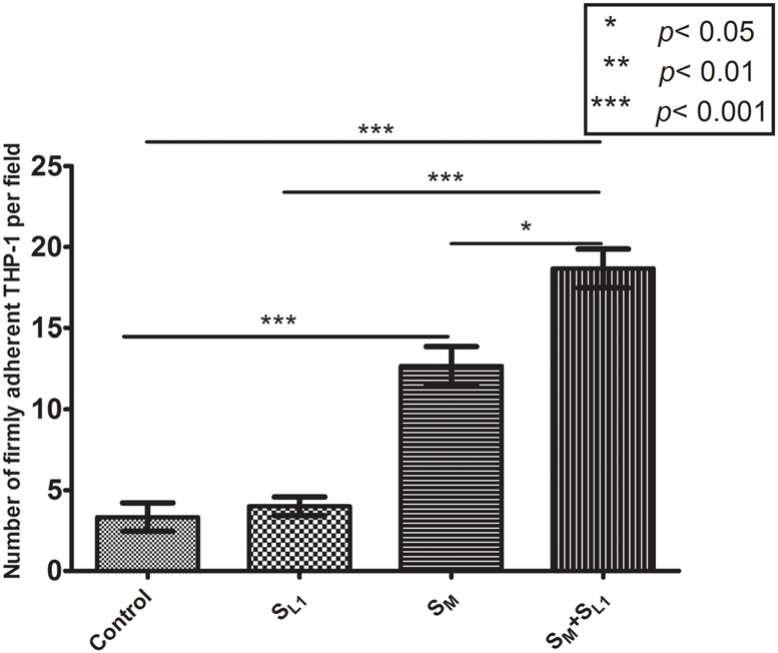
Number of firmly adherent THP-1 cells on resting or activated HUVEC under shear flow conditions, according to micrfluidic channel-based detachment assays. Here, “Control”—resting endothelium, “S_L1_“—the supernatant of 8 μg/ml oxLDL-treated mast cells, “S_M_“—the supernatant of 8 μg/ml oxLDL-treated macrophages, and “S_M_ + S_L1_“—the combination of the supernatants of the oxLDL-treated macrophages and mast cells. Mean μ SD of three independent tests. More details about the HUVEC activation groups are given in Figs. 1 and 7.

Fig. 8 depicts the data on the firm adhesion of THP-1 cells to HUVEC under shear flow conditions. In the control group and the S_L1_ activation group, the number of firmly adherent THP-1 cells was 3±1 and 4±1, respectively. This population was significantly (*p* < 0.001) increased to 13±1, when HUVEC were activated by S_M,_ and further significantly (*p* < 0.05) increased to 19±1 with the combined S_M_ +S_L1_ exposure_._

**Fig 9:**
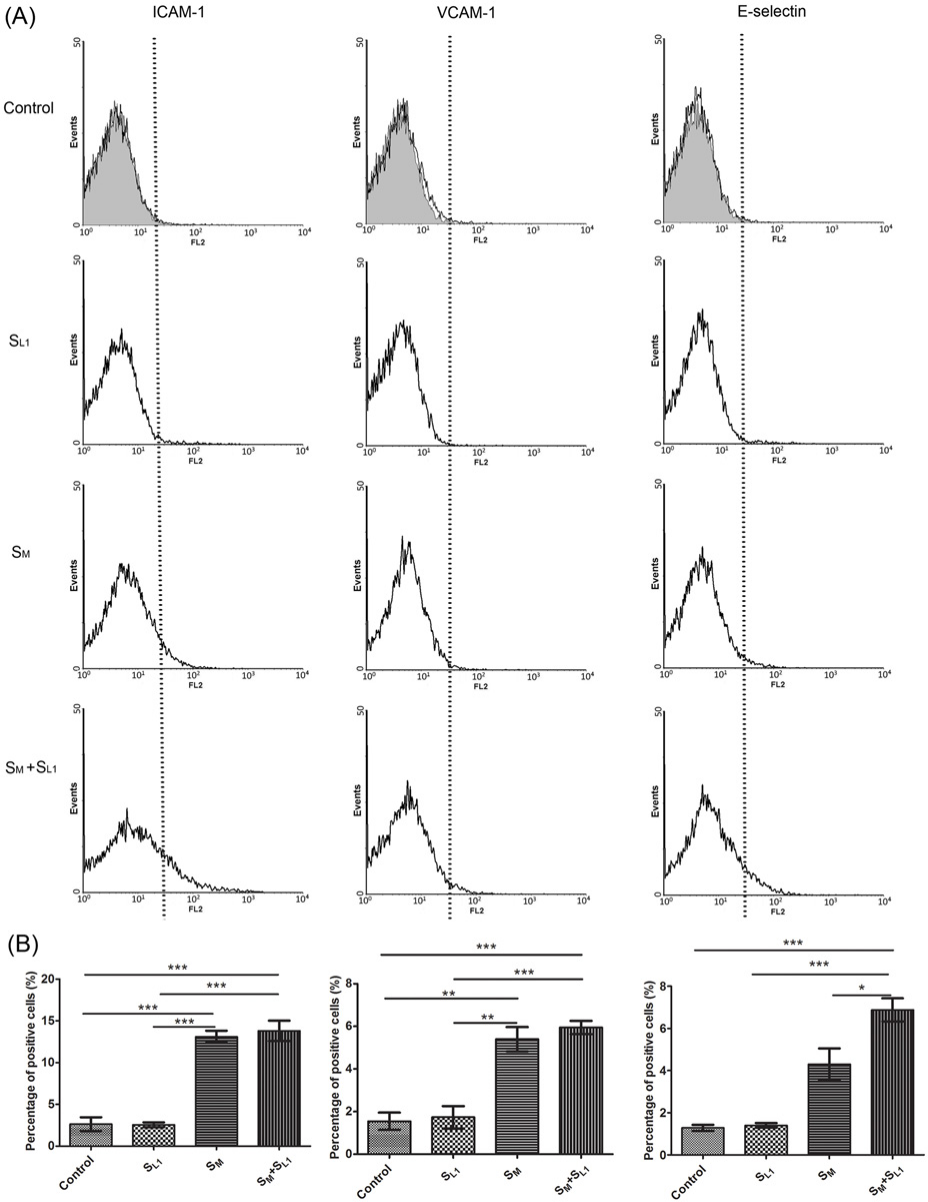
Surface expression of adhesion molecules on resting HUVEC (“Control”) and on HUVEC exposed to the supernatant of 8 μg/ml oxLDL-treated mast cells (“S_L1_”), the supernatant of 8 μg/ml oxLDL-treated macrophages (“S_M_”), or a combination of these supernatants (S_M_ + S_L1_). (A) Histogram of the expression of ICAM-1, VCAM-1 and E-selectin on the HUVEC surface; (B) Percentage of HUVEC with positive expression of these adhesion molecules (mean ± SD, n = 3). * *p* < 0.05, ** *p* < 0.01, *** *p* < 0.001. Details about the HUVEC activation groups are given in Figs. 1 and 7.

Flow cytometric measurements of ICAM-1, VCAM-1 and E-selectin expression on the endothelial cell surface in the control, S_M_, S_L1_, and S_M_ + S_L1_ groups are shown in Fig. 9. According to the histograms (Fig. 9A), the S_M_ + S_L1_ activation had the most effect on the expression of endothelial cell adhesion molecules. The percentage of endothelial cells positively expressed ICAM-1, VCAM-1 and E-selectin significantly (*p*<0.001) increased from 2.6%±0.8%, 1.6%±0.7% and 1.3%±0.3% in the control group to 13.8%±1.2%, 5.9%±0.5% and 6.9%±0.5% in the S_M_ + S_L1_ group, respectively (Fig. 9B). The S_L1_ alone activation had a statistically insignificant effect on the expression of all of these molecules: 2.6%±0.3% for ICAM-1, 1.7%±0.9% for VCAM-1, and 1.4%±0.2% for E-selectin. The S_M_ alone activation significantly (*p* < 0.01) increased the percentage of HUVEC with ICAM-1 (13.1%±1.3%) and VCAM-1 (5.4%±0.6%) but not with E-selectin (4.3%±0.8%). These results are similar to those shown in Fig. 5, where the supernatant of oxLDL-treated macrophages in combination with histamine but not the supernatant alone had a statistical significant effect on the E-selectin expression. The presence of E-selectin enhances leukocyte rolling and crawling [43, 44] and triggers β2 integrin (including LFA-1 and Mac-1 integrin) binding to ICAM-1 [45, 46]. The binding of ICAM-1 to β2 integrin generates the bond force that drives monocyte firm adhesion to vascular endothelial cells. Thus, the increased density of E-selectin on the surface of endothelial cells increases the number of crawling cells that will eventually arrest on the endothelium. This may explain why flow assays predict a higher ratio of adherent THP-1 cells between the S_M_ + S_L1_ and S_M_ activation groups (1.46) than static assays (1.27). In summary, our data show that the increased expression of E-selectin may be behind the synergistic increase of monocyte-endothelium adhesion by inflammatory cytokines from oxLDL-treated macrophages and mast cells.

## Discussion

LDL particles are produced in bloodstream from very low density lipoprotein (VLDL) particles that are continuously losing triacylglycerols [47]. They transports cholesterol to cells in the artery wall, and when the plasma cholesterol concentration increases above a certain limit (150 mg/dL), a risk for atheromatous plaque development significantly increases [4, 48, 49]. Lipid rich diet is a main source of LDL in the body because the blood cholesterol and LDL levels are reduced with reducing dietary saturated fat and cholesterol [50, 51].

When circulating LDL particles enter the tunica intima, they become exposed to oxidants derived from endothelial cells, macrophages, and smooth muscle cells [52–54] and undergo oxidative modification. Depending on the LDL and oxidant concentrations, this process may result in either minimally modified/oxidized LDL (mmLDL), which lipids but not proteins are modified, or oxLDL containing modified lipids and proteins [55]. OxLDL but not LDL or mmLDL binds to scavenger receptors on endothelial cells and macrophages, leading to the endothelial cell activation via CD40/CD40L signaling pathway [9] and the release of TNF-α from and foam cell transformation of macrophages [2, 15]. This suggests two major ways by which oxLDL particles cause endothelial dysfunction in the earliest stage of atherosclerotic plaque development: 1) direct interaction with endothelial cells, and 2) release of pro-inflammatory cytokines from macrophages and other cells present in the artery wall. We have analyzed both these scenarios and found that endothelial cells are much more responsive to cytokines from oxLDL-activated tissue-resident cells than to oxLDL itself. For example, the endothelium exposed to the supernatants of low dose (8 μg/ml) oxLDL-treated cells had much more firmly adherent monocytes than the endothelium directly stimulated with a high dose (80 μg/ml) of oxLDL. Thus, scavenger receptors on tissue-resident cells or the endothelial receptors for pro-inflammatory cytokines released by activated tissue-resident cells should be the focus of preventive medicine against atherosclerosis.

The other important result of our work is that oxLDL can co-activate at least two types of tissue-resident cells—macrophages and mast cells. This co-activation induces macrophages and mast cells to release two powerful pro-inflammatory mediators, TNF-α and histamine. As a pro-inflammatory mediator, TNF-α triggers nuclear factor kappa B (NF-κB) pathway and is able to induce the expression of several inflammatory cytokines and chemokines by endothelial cells, such as interleukin-1α (IL-1α), interleukin-8 (IL-8) and chemoattractant protein-1 (MCP-1) [56–59]. Both IL-1α and IL-8 actively participate in endothelial activation, while MCP-1 is a major chemotactic factor for monocytes and macrophages [60, 61]. Therefore, endothelial cells can be directly or indirectly activated by TNF-α. Furthermore, as we showed earlier [38], TNF-α and histamine have a synergistic effect on endothelial dysfunction and monocyte adhesion. Specifically, histamine applied to TNF-α-activated endothelium significantly increases the surface expression of endothelial E-selectin and the number of firmly adherent monocytes. E-selectin plays a pivotal role in transition from cell rolling to crawling and finally to firm adhesion [44–46]. Due to the increased expression of endothelial E-selectin, the synergistic effect of TNF-α and histamine on monocyte-endothelium adhesion becomes particularly strong under shear flow conditions.

Mast cells have been recognized as a key player in advanced atherosclerosis [62, 63], and recent in vivo studies show that they also are important in atherogenesis [64]. However, the mechanism by which they become involved in this process is not established. Mast cells reside in the perivascular or adventitial tissue of healthy blood vessels, and they can adhere to the atheroslerotic lesion surface or to the extracellular matrix via adhesion molecules [65]. Thus, in early atherosclerosis, they are not in the closest proximity to macrophages in the intima, and, as such, cannot directly interact with these cells. The results of our work suggest that adventitial mast cells are initially activated by oxLDL particles transported from the intima to the adventitia. This leads to the release of histamine, which then diffuse from the adventitia to the intima and enhances the endothelial dysfunction caused by TNF-α from activated intimal macrophages. Previously, it was shown that the secretion of histamine from mast cells can be induced by OxLDL-IgG immune complexes [25], but here we showed that even a low dose of oxLDL directly applied to mast cells is sufficient to produce histamine at the level at which the monocyte adhesion to endothelium will be substantially increased.

Later on, mast cells migrate into the lesion, most likely with the help of chemokines, e.g. transforming growth factor-beta (TGF-β) [66] secreted by macrophages [67]. This leads to direct interactions between macrophages and mast cells that further speed up the plaque formation and cause the degradation of extracellular matrix proteins in the shoulder region of the vulnerable plaque [17, 68–70]. The investigation of the mechanisms responsible for mast cell migration and mast cell-macrophage interactions in atherosclerosis will be the topic of our future studies.

Holvoet et al. [37] have shown that the concentration of oxLDL in human body ranges from 6 μg/ml to 33 μg/ml. The upper limit of this range is still much less than the oxLDL concentration that we used for the direct activation of vascular endothelium. It has been reported that coronary artery disease (CAD) patients have the circulating oxLDL concentration of more than 15 μg/ml, while the patients with a lower concentration of oxLDL show no obvious evidence of CAD [37]. Even without the symptoms, these patients may still have fatty streaks formed in their vessels as a result of pro-inflammatory mediators released from macrophages and mast cells activated by oxLDL. The chronic inflammation induced by these mediators may continue for years, with no clinical signs, eventually progressing into atheroma [48]. Our study shows that oxLDL with a concentration as low as 8 μg/ml is able to activate macrophages and mast cells to respectively release TNF-α and histamine, thus enhancing monocyte recruitment. This observation points out to a mechanism by which low-dose OxLDL induces the accumulation of monocytes/macrophages in the intimal layer of the artery.

The degranulation of mast cells during allergy attacks may lead to spikes in the histamine concentration near or inside atherosclerotic lesions. The amount of released histamine can be much more than that produced by oxLDL-activated mast cells. Thus, allergy attacks can further weaken the endothelium already exposed to TNF-α and histamine from oxLDL-activated macrophages and mast cells. This leads to our final conclusion: patients with allergy or asthma who have an elevated amount of histamine in tissues are at very high risk for atherosclerosis and cardiovascular disease if they also have a lipid-rich diet. In fact, recent clinical data [71] point out that allergy and asthma contribute to the development of atherosclerosis.
